# Diabetic and stress‐induced hyperglycemia in spontaneous intracerebral hemorrhage: A multicenter prospective cohort (CHEERY) study

**DOI:** 10.1111/cns.14033

**Published:** 2022-11-29

**Authors:** Shaoli Chen, Yan Wan, Hongxiu Guo, Jing Shen, Man Li, Yuanpeng Xia, Lei Zhang, Zhou Sun, Xiaolu Chen, Gang Li, Quanwei He, Bo Hu

**Affiliations:** ^1^ Department of Neurology, Union Hospital, Tongji Medical College Huazhong University of Science and Technology Wuhan Hubei Province China; ^2^ Department of Neurology the First Affiliated Hospital of Shihezi University Medical College Shihezi Xinjiang Province China

**Keywords:** diabetes mellitus, hyperglycemia, intracerebral hemorrhage, stress‐induced hyperglycemia; prognosis

## Abstract

**Introduction:**

Admission hyperglycemia is a common finding after spontaneous intracerebral hemorrhage (ICH) secondary to pre‐existing diabetes mellitus (DM) or stress‐induced hyperglycemia (SIH). Studies of the causal relationship between SIH and ICH outcomes are rare.

**Aim:**

We aimed to identify whether SIH or pre‐existing DM was the cause of admission hyperglycemia associated with ICH outcomes.

**Methods:**

Admission glycosylated hemoglobin (HbA1c), glucose levels, and comorbidity data from the prospective, multicenter cohort, Chinese Cerebral Hemorrhage: Mechanisms and Intervention Study (CHEERY), were collected and analyzed. According to different admission blood glucose and HbA1c levels, patients were divided into nondiabetic normoglycemia (NDN), diabetic normoglycemia (DN), diabetic hyperglycemia (DH), and SIH groups. Modified Poisson regression models were used to analyze ICH outcomes in the different groups.

**Results:**

In total, 1372 patients were included: 388 patients with admission hyperglycemia, 239 with DH, and 149 with SIH. In patients with hyperglycemia, SIH was associated with a higher risk of pulmonary infection [risk ratios (RR): 1.477, 95% confidence interval (CI): 1.004–2.172], 30‐day (RR: 1.068, 95% CI: 1.009–1.130) and 90‐day mortality after ICH (RR: 1.060, 95% CI: 1.000–1.124).

**Conclusions:**

Admission hyperglycemia is a common finding after ICH, and SIH is a sensitive predictor of the risk of pulmonary infection and all‐cause death after ICH.

## Graphical abstract

In patients with hyperglycemia, stress‐induced hyperglycemia (SIH) was associated with a higher risk of pulmonary infection [risk ratios (RR): 1.477, 95% confidence interval (CI): 1.004–2.172] and 30‐day (RR: 1.068, 95% CI: 1.009–1.130) and 90‐day mortality after intracerebral hemorrhage (ICH) (RR: 1.060, 95% CI: 1.000–1.124). Stress‐induced hyperglycemia is a sensitive predictor of the risk of pulmonary infection and all‐cause death after ICH.

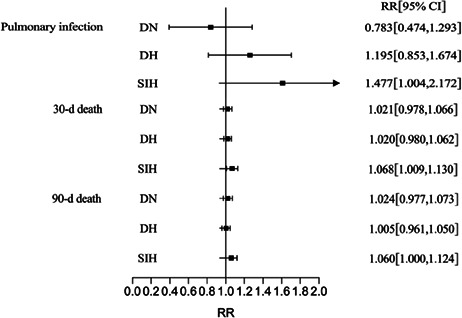

## 1 INTRODUCTION

Spontaneous intracerebral hemorrhage (ICH) is the second most common and fatal type of stroke,^1^ with a 30‐day mortality rate of up to 40%.^2^ Hyperglycemia is always observed at admission when one suffers from ICH and is considered a predictor of poor outcomes in some studies,^3^, ^4^, ^5^, ^6^, ^7^ which were challenged by other studies.^8^, ^9^, ^10^ This discrepancy may be due to the causes of admission hyperglycemia, which could either be stress‐induced hyperglycemia (SIH) or pre‐existing diabetes mellitus (DM), which were not well differentiated in those studies. SIH is a transient hyperglycemic condition caused by acute diseases and is usually restricted to patients without DM,^11^ and is defined as admission blood glucose ≥7.8 mmol/L,^12^ which has been found to be directly responsive to the severity and predictable to the poor outcomes of ICH.^13^, ^14^, ^15^, ^16^ However, there are still some shortcomings in these well‐designed reports: (1) up to 1/3 of patients with occult diabetes may clutter the results^17^ as some previous studies defined SIH in non‐DM patients only by disease history and not by measuring glycosylated hemoglobin A1c (HbA1c),^15^, ^16^, ^18^ a reliable measure of the mean glucose concentration over the previous 3‐ to 4‐month time period^19^, ^20^; (2) the few studies pertaining to SIH in patients with ICH were all relatively small populations (the largest included 328 patients)^9^, ^13^, ^14^, ^16^; and (3) some were retrospective studies.^14^

Therefore, we sought to determine whether SIH or pre‐existing DM was associated with poor outcomes of ICH in a large, multicentric, prospective cohort of patients with ICH having a long follow‐up.

## 2 METHODS

### 2.1 Participants and design

We analyzed data from the Chinese Cerebral Hemorrhage: Mechanisms and Intervention study (CHEERY) (registration number of China Clinical Trial Registration Center: ChiCTR1900020872, http://www.chictr.org.cn). The study protocol and data collection were conducted in strict accordance with the Declaration of Helsinki and approved by the Research Ethics Committee of Tongji Medical College, Huazhong University of Science and Technology, Wuhan, China (ethical approval number: 2018‐S485). All patients provided informed consent before recruitment. Consecutive patients presenting with spontaneous ICH were admitted to 31 hospital centers between December 2018 and June 2021. Patients aged ≥18 years, with spontaneous ICH confirmed using computed tomography (CT), and within 24 h of onset were recruited for this study. Patients who met the following criteria were excluded: (1) hemorrhages secondary to trauma, primary subarachnoid hemorrhage, hemorrhagic conversion from ischemic stroke, and thrombolysis; (2) lack of data on admission glucose or HbA1c levels; and (3) unavailability of imaging and baseline information.

### 2.2 Data collection and follow‐up

Relevant information was collected through the electronic medical record system: age, sex, disease history, admission systolic blood pressure (SBP), admission blood glucose, HbA1c level, time from symptom onset to admission, and surgical treatment. Admission blood glucose and HbA1c were measured after an overnight 8‐h fast. Baseline neurological deficits were assessed using the Glasgow Coma Scale (GCS).^21^ Hematoma localization and intraventricular hemorrhage (IVH) were recorded according to the first head CT data after admission, and hematoma volume was calculated using the ABC/2 formula.^22^, ^23^ Pulmonary infection was diagnosed by two well‐trained and experienced neurologists according to the modified Centers for Disease Control and Prevention criteria, in combination with the patient's clinical symptoms, laboratory, and radiological examinations within 1 week after ICH.^24^, ^25^ Stroke neurologists conducted face‐to‐face or telephonic interviews with the enrolled patients on day 30 and 90 after the onset of ICH. The degree of functional recovery was evaluated according to a modified Rankin Scale (mRS) score,^23^ and poor outcome was defined as mRS score of 3–6.^26^, ^27^, ^28^

### 2.3 Definition of subgroup

According to the latest consensus from the American Association of Clinical Endocrinologists and American Diabetes Association,^12^ SIH was defined as having no DM history, HbA1c <6.5%, and admission blood glucose ≥7.8 mmol/L. If the admission blood glucose <7.8 mmol/L, it was defined as nondiabetic normoglycemia (NDN). Diabetic hyperglycemia (DH) was defined as having a DM history or HbA1c ≥6.5%, and admission blood glucose ≥7.8 mmol/L; and if admission blood glucose <7.8 mmol/L, it was defined as diabetic normoglycemia (DN).

### 2.4 Statistical analysis

SPSS statistical software (version 26.0, SPSS Corporation, Chicago) was used to analyze the data, and the statistical significance was set at *p* < 0.05. Categorical variables are expressed as percentages, and the χ^2^ test or Fisher's exact test was used to compare the differences between groups. The Kolmogorov–Smirnov test (KS test) for normality was used to assess the data distribution of continuous variables. Normally distributed variables are expressed as mean ± standard deviation (SD), and two groups were compared using Student's *t*‐test. Non‐normally distributed variables were expressed as median and interquartile ranges (first and third quartiles), and the Mann–Whitney U test or Kruskal–Wallis H test was used to compare differences between groups. In univariate analysis, variables reaching *p* < 0.05 were considered to have significant differences. Finally, age, sex, history of hypertension, time from symptom onset to admission, infratentorial hemorrhage, hematoma volume, GCS, IVH, and surgical treatment were included in the regression model in the multivariate analysis. Modified Poisson regression models were used to calculate risk ratios (RRs)^29^ and the associated 95% confidence intervals (CIs) for the association between different groups and the outcomes of interest, and figures were drawn using GraphPad Prism 9.0.

## 3 RESULTS

### 3.1 Patient characteristics

**FIGURE 1:**
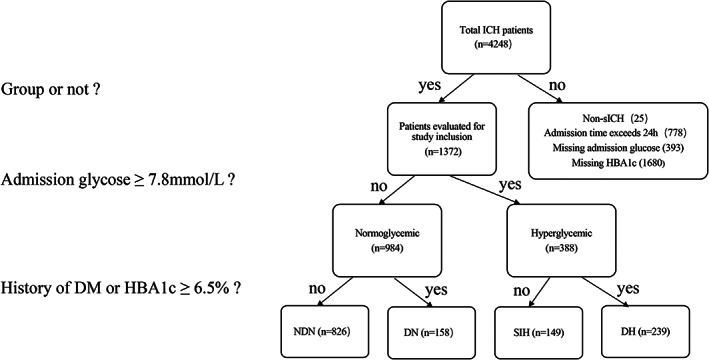
Breakdown of the study population with identification of four groups of patients based on admission blood glucose, HbA1c, and history of DM. DH, diabetic hyperglycemia; DN, diabetic normoglycemia; NDN, nondiabetic normoglycemia; SIH, stress‐induced hyperglycemia.

From December 2018 to June 2021, 4248 patients with ICH were enrolled in the CHEERY study. After excluding 25 patients with non‐spontaneous ICH, 778 patients who presented with symptoms exceeding 24 h, 393 patients who lacked admission blood glucose data, and 1680 patients with missing HbA1c data, a total of 1372 patients with complete study data were finally included in the analysis. Among them, 984 (71.7%) patients presented with normoglycemia, and 388 (28.3%) had a hyperglycemic status on admission. According to the definitions for the different glucose classifications mentioned above, 826 patients were classified as having NDN, 158 patients were classified as having DN, 149 patients were classified as having SIH, and 239 patients were classified as having DH (Figure 1).

### 3.2 Characteristics and outcomes of patients with different admission blood glucose levels

The baseline characteristics and outcomes of ICH patients with hyperglycemia or normoglycemia are compared in Table 1. The mean age of the included patients was 62.3 ± 11.6 years, 904 (65.9%) patients were male, and 281 (20.5%) patients had a history of DM. The average age, sex ratio, admission SBP, hematoma volume, and length of hospitalization were not significantly different between the hyperglycemic and normoglycemic groups. In the hyperglycemia group, the median of admission blood glucose was 9.5 (8.5–11.5) mmol/L, and the mean HbA1c was 6.4%, both of which were higher than those in the normoglycemic group (*p* < 0.001). Patients with hyperglycemia were more likely to have hypertension (76.5% vs. 67.1%, *p* = 0.002), DM (47.2% vs. 10.0%, *p* < 0.001), shorter time from symptom onset (3 vs. 3.5, *p* = 0.012), infratentorial hemorrhage (18.3% vs. 9.5%, *p* < 0.001), IVH (22.9% vs. 12.0%, *p* < 0.001), lower GCS score (13 vs. 14, *p* < 0.001), more frequent surgical treatment (26% vs. 13.1%, *p* < 0.001), and higher risk of pulmonary infection (20.1% vs. 10.3%, *p* < 0.001). In addition, patients with hyperglycemia were more likely to have poor outcomes, which were defined as mRS 3–6, on day 30 (72.5% vs. 57.4%, *p* < 0.001) and 90 (63.2% vs. 46.2%, *p* < 0.001), as well as higher risk of mortality during hospitalization (6.4% vs. 2.3%, *p* < 0.001) and 30 days(21.4% vs. 7.8%, *p* < 0.001) and 90 days (23.8% vs. 11.1%, *p* < 0.001) after ICH onset compared with those with normoglycemia.

**TABLE 1:** Baseline characteristics and outcomes of ICH patients with different admission blood glucose levels

	All subjects (*N* = 1372)	Admission blood glucose <7.8 (*N* = 984)	Admission blood glucose ≥7.8 (*N* = 388)	*p*‐value
Age, y, Mean ± SD	62.3 ± 11.6	62.2 ± 11.5	62.6 ± 11.9	0.158
Male, (*n*, %)	904 (65.9)	651 (66.2)	253 (65.2)	0.738
Hypertension, (*n*, %)	957 (69.8)	660 (67.1)	297 (76.5)	0.002
DM, (*n*, %)	281 (20.5)	98 (10.0)	183 (47.2)	<0.001
Time from symptom onset to admission, h, median (IQR)	3 (2, 7.5)	3.5 (2, 8)	3 (2, 6)	0.012
Admission SBP ≥140 mmHg, (*n*, %)	1219 (88.8)	869 (88.3)	350 (90.2)	0.316
HBA1c ≥6.5%, (*n*, %)	397 (28.9)	158 (16.1)	239 (61.6)	<0.001
Admission blood glucose, mmol/L, median (IQR)	6.5 (5.5, 8.1)	5.9 (5.3, 6.6)	9.5 (8.5, 11.5)	<0.001
HBA1c, %, median (IQR)	5.7 (5.3, 6.2)	5.6 (5.3, 6.0)	6.4 (5.6, 8.0)	<0.001
Infratentorial hemorrhage, (*n*, %)	159 (12.0)	90 (9.5)	69 (18.3)	<0.001
Hematoma volume, ml, median (IQR)	10.0 (4.6, 24.0)	10.0 (4.5, 20.0)	10.0 (4.3, 27.0)	0.124
IVH, (*n*, %)	207 (15.1)	118 (12.0)	89 (22.9)	<0.001
GCS, median (IQR)	14 (12, 15)	14 (13, 15)	13 (9, 15)	<0.001
Surgical treatment, (*n*, %)	230 (16.8)	129 (13.1)	101 (26%)	<0.001
Length of hospitalization, days, median (IQR)	16.0 (11.0, 22.0)	16.0 (12.0, 21.0)	16.0 (9.0, 23.0)	0.685
Pulmonary infection, (*n*, %)	179 (13.0)	101 (10.3)	78 (20.1)	<0.001
30‐day mRS (3–6), (*n*, %)	821/1331^a^ (62.7)	547/953^a^ (57.4)	274/378^a^ (72.5)	<0.001
90‐day mRS (3–6), (*n*, %)	679/1331^a^ (51.0)	440/953^a^ (46.2)	239/378^a^ (63.2)	<0.001
In‐hospital death, (*n*, %)	48 (3.5)	23 (2.3)	25 (6.4)	<0.001
30‐day death, (*n*, %)	155/1331^a^ (11.6)	74/953^a^ (7.8)	81/378^a^ (21.4)	<0.001
90‐day death, (*n*, %)	196/1331^a^ (14.7)	106/953^a^ (11.1)	90/378^a^ (23.8)	<0.001

Abbreviations: DM, diabetes mellitus; GCS, Glasgow Coma Scale; HbA1c, Glycosylated hemoglobin A1c; IQR indicates interquartile range; IVH, intraventricular hemorrhage; mRS, modified Rankin Scale; SBP, systolic blood pressure; SD, standard deviation.

^a^ 41 patients lacked 30‐ and 90‐days follow‐up data, of which 31 with non‐hyperglycemia and 10 with hyperglycemia.

### 3.3 Multivariate analysis for association between hyperglycemia and patient outcomes

The multivariate analysis of the association between hyperglycemia and ICH outcomes is shown in Table 2. The following variables were used in the adjusted models: age, sex, history of hypertension, time from symptom onset to admission, infratentorial hemorrhage, IVH, GCS, and surgical treatment. The results revealed that hyperglycemia was independently associated with increased risk of pulmonary infection (RR: 1.403, 95% CI: 1.066–1.846), poor 30‐day (RR: 1.038, 95% CI: 1.003–1.074) and 90‐day outcomes (RR: 1.145, 95% CI: 1.025–1.279), and 30‐day mortality due to ICH (RR: 1.041, 95% CI 1.005–1.077), while hyperglycemia was not associated with mortality during hospitalization and 90 days after ICH (Table 2).

**TABLE 2:** Multivariate regression analysis of outcomes of hyperglycemic patients

	Admission blood glucose ≥7.8		
	RR	95% CI	*p* value
Pulmonary infection	1.403	1.066–1.846	0.016
30‐day mRS (3–6)	1.038	1.003–1.074	0.034
90‐day mRS (3–6)	1.145	1.025–1.279	0.016
In‐hospital death	1.138	0.577–2.242	0.710
30‐day death	1.041	1.005–1.077	0.025
90‐day death	1.029	0.991–1.069	0.132

*Note*: Model: age, sex, hypertension history, time from symptom onset to admission, infratentorial hemorrhage, IVH, GCS, surgical treatment.

Abbreviations: CI indicates confidence interval; GCS, Glasgow Coma Scale; IVH, intraventricular hemorrhage; mRS, modified Rankin Scale; RR, Relative Risk.

### 3.4 Characteristics and outcomes of ICH patients with different glucose classifications

Hyperglycemia at admission may be caused by SIH or DM. According to the HbA1c level, glucose level, and DM history, patients were classified into four groups: NDN, DN, DH, and SIH, as previously mentioned. The characteristics and outcomes of the four classifications are presented in Table 3. Overall, no significant differences in sex (*p* = 0.446) and age (*p* = 0.353) among the four groups were found. Compared with the other groups, patients with DH had a significantly larger hematoma volume (*p* < 0.001) and a higher incidence of IVH (*p* < 0.001). Patients with SIH had a significantly higher incidence of infratentorial ICH (*p* < 0.001), lower GCS score (*p* < 0.001), and a higher proportion of surgical treatment (*p* < 0.001) and higher risk of pulmonary infection (*p* < 0.001). In addition, patients with SIH were more likely to have poor outcomes (mRS 3–6) at 30 and 90 days after ICH (p < 0.001), and the risk of their death was higher during hospitalization, at 30 and 90 days after ICH, than the other groups (*p* < 0.001).

**TABLE 3:** Baseline characteristics and outcomes of patients with different glucose classifications

	Admission blood glucose<7.8		Admission blood glucose ≥7.8		*p* value
	NDN (*N* = 826)	DN (*N* = 158)	DH (*N* = 239)	SIH (*N* = 149)	
Age, y, Mean ± SD	62.0 ± 11.6	63.1 ± 10.8	63.2 ± 11.5	61.6 ± 12.4	0.353
Male, (*n*, %)	545 (66.0)	106 (67.1)	163 (68.2)	90 (60.4)	0.446
Hypertension, (*n*, %)	529 (64.0)	131 (82.9)	188 (78.7)	109 (73.2)	<0.001
DM, (*n*, %)	0 (0)	98 (62.0)	183 (76.6)	0 (0)	<0.001
Time from symptom onset to admission (h), median (IQR)	3.5 (2, 8)	4 (2, 10.5)	3 (2, 7)	3 (2, 5)	0.038
Admission SBP ≥140 mmHg, (*n*, %)	731 (88.5)	138 (87.3)	220 (92.1)	130 (87.2)	0.345
Admission blood glucose, mmol/L, median (IQR)	5.9 (5.2, 6.5)	6.4 (5.4, 7.0)	10.8 (9.1, 13.8)	8.8 (8.3, 9.9)	<0.001
HBA1c, %, median (IQR)	5.6 (5.2, 5.9)	6.7 (6.5, 7.4)	7.8 (6.8, 9.1)	5.6 (5.2, 6.0)	<0.001
Infratentorial hemorrhage, (*n*, %)	78 (9.8)	12 (7.7)	36 (15.4)	33 (23.1)	<0.001
Hematoma volume, (ml), median (IQR)	10.0 (4.1, 20.0)	10.0 (5.0, 28.9)	12.8 (5.0, 35.0)	11.8 (4.9, 30.5)	<0.001
IVH, (*n*, %)	98 (11.9)	20 (12.7)	58 (24.3)	31 (20.8)	<0.001
GCS, median (IQR)	14 (13, 15)	14 (11, 15)	13 (9, 15)	12 (7, 15)	<0.001
Surgical treatment, (*n*, %)	103 (12.5)	26 (16.5)	61 (25.5)	40 (26.8)	<0.001
Length of hospitalization, (days), median (IQR)	17.0 (12.0, 22.0)	15.0 (11.0, 20.0)	17.0 (9.8, 26.0)	16.0 (7.3, 21.0)	0.051
Pulmonary infection, (*n*, %)	84 (10.2)	17 (10.8)	46 (19.2)	32 (21.5)	<0.001
30‐day mRS (3–6), (*n*, %)	457/799 (57.2)	90/154 (58.4)	169/235 (71.9)	105/143 (73.4)	<0.001
90‐day mRS (3–6), (*n*, %)	364/799 (45.2)	76/154 (50.0)	147/235 (63.4)	92/143 (64.3)	<0.001
In‐hospital death, (*n*, %)	15 (1.8)	8 (5.1)	14 (5.9)	11 (7.4)	<0.001
30‐day death, (*n*, %)	51/799 (6.4)	23/154 (14.9)	43/235 (18.3)	38/143 (26.6)	<0.001
90‐day death, (*n*, %)	78/799 (9.8)	28/154 (18.2)	48/235 (20.4)	42/143 (29.4)	<0.001

Abbreviations: DH, diabetic hyperglycemia; DM, diabetes mellitus; DN, diabetic normoglycemia; GCS, Glasgow Coma Scale; IQR, interquartile range; IVH, intraventricular hemorrhage; mRS, modified Rankin Scale; NDN, nondiabetic normoglycemia; SBP, systolic blood pressure; SD, standard deviation; SIH, stress‐induced hyperglycemia.

### 3.5 Multivariate analysis for outcomes of patients with ICH and different glucose classifications

The results of the multivariate regression models (adjusted for age, sex, hypertension history, time from symptom onset to admission, infratentorial hemorrhage, hematoma volume, GCS, IVH, and surgical treatment) are shown in Table 4. Compared with patients with NDN, the risks of poor outcomes and death in patients with DN and DH did not increase.

**TABLE 4:** Multivariate regression analysis of outcomes of patients with different glucose classifications

RR (95% CI)	NDN	DN	*p* value	DH	*p* value	SIH	*p* value
Pulmonary infection	Ref	0.783 (0.474, 1.293)	0.339	1.195 (0.853, 1.674)	0.301	1.477 (1.004, 2.172)	0.047
30‐day mRS (3–6)	Ref	0.974 (0.924, 1.028)	0.341	1.022 (0.981, 1.065)	0.299	1.036 (0.987, 1.088)	0.155
90‐day mRS (3–6)	Ref	1.011 (0.846, 1.207)	0.907	1.118 (0.979, 1.277)	0.101	1.136 (0.975, 1.324)	0.103
In‐hospital death	Ref	1.435 (0.593, 3.474)	0.424	1.177 (0.513, 2.701)	0.701	1.251 (0.506, 3.092)	0.628
30‐day death	Ref	1.021 (0.978, 1.066)	0.343	1.020 (0.980, 1.062)	0.340	1.068 (1.009, 1.130)	0.022
90‐day death	Ref	1.024 (0.977, 1.073)	0.332	1.005 (0.961, 1.050)	0.838	1.060 (1.000, 1.124)	0.049

*Note*: Model: age, sex, hypertension history, time from symptom onset to admission, infratentorial hemorrhage, hematoma volume, GCS, IVH, surgical treatment.

Abbreviations: CI indicates confidence interval; DH, diabetic hyperglycemia; DN, diabetic normoglycemia; GCS, Glasgow Coma Scale; IVH, intraventricular hemorrhage; mRS, modified Rankin Scale; NDN, nondiabetic normoglycemia; RR, Relative Risk; SIH, stress‐induced hyperglycemia.

**FIGURE 2:**
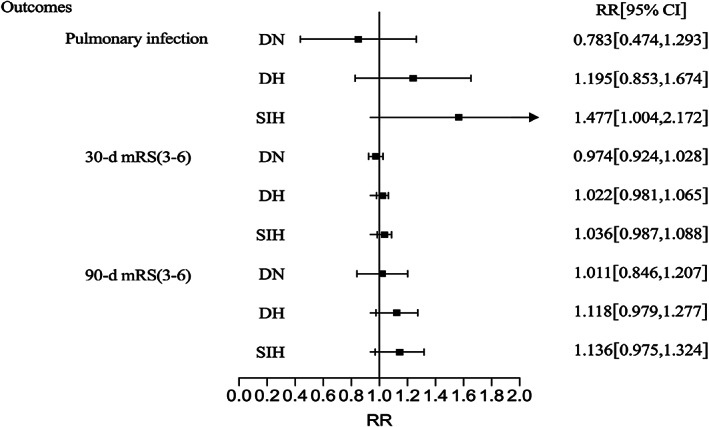
Multivariate regression analysis of ICH poor outcomes of different subgroups. Adjusted: Adjusted for age, sex, time from symptom onset to admission, infratentorial hemorrhage, hematoma volume, GCS, IVH, hypertension history, and surgical treatment. DH, diabetic hyperglycemia; DN, diabetic normoglycemia; SIH, stress‐induced hyperglycemia.

**FIGURE 3:**
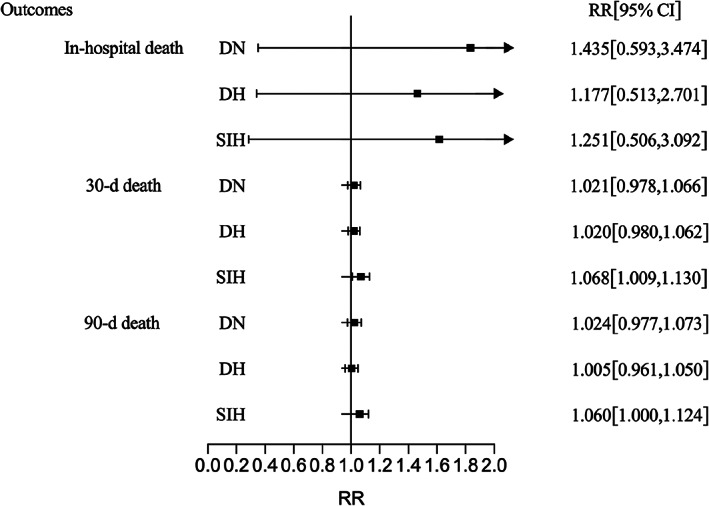
Multivariate regression analysis of ICH mortality of different subgroups. Adjusted: Adjusted for age, sex, time from symptom onset to admission, infratentorial hemorrhage, hematoma volume, GCS, IVH, hypertension history, and surgical treatment. DH, diabetic hyperglycemia; DN, diabetic normoglycemia; SIH, stress‐induced hyperglycemia.

Patients with SIH had a higher risk of pulmonary infection (RR: 1.477, 95% CI: 1.004–2.172), 30‐ (RR: 1.068, 95% CI: 1.009–1.130) and 90‐day mortality (RR: 1.060, 95% CI: 1.000–1.124) (Figure 2 and 3 **)**.

## 4 DISCUSSION

In this study, we found that hyperglycemia was associated with poor prognosis and an increased risk of death after ICH onset. After multivariate regression analysis, admission hyperglycemia was an independent risk factor for pulmonary infection, poor 30‐ and 90‐day prognosis, and 30‐day mortality but did not increase the risk of 90‐day mortality. Compared with patients with NDN, DH did not increase the risk of poor outcome and mortality, whereas SIH was an independent risk factor for pulmonary infection and 30‐ and 90‐day death after ICH.

Previous studies have shown that ICH is often accompanied by hyperglycemia, and the association between admission hyperglycemia and the risk of death and adverse outcomes of ICH has been concerning.^3^, ^4^, ^5^, ^6^, ^7^, ^8^, ^9^, ^10^ Hyperglycemia leads to peripheral nerve injury,^30^ and hematoma perihematomal cell death^31^ and decreased autophagy,^32^ increasing the production of superoxide caused by tissue plasminogen activator,^33^ and increasing the plasma kallikrein to promote the expansion of hematoma.^34^ These processes may be the causes of hyperglycemia, which aggravates the poor outcomes of ICH. The results of INTERACT2 showed that hyperglycemia and DM were independent predictors of poor prognosis in patients with mild‐ to moderate–severe ICH,^7^ and a considerable number of studies have shown that admission hyperglycemia is associated with poor outcomes and death risk after ICH.^3^, ^8^, ^10^, ^35^, ^36^, ^37^, ^38^, ^39^, ^40^ In addition, our study revealed that admission hyperglycemia was associated with an increased risk of poor 30‐ and 90‐day poor outcomes and 30‐day mortality after ICH.

However, contradictions remain. Tetri et al. found that admission hyperglycemia was not an independent risk factor for the prognosis of ICH.^8^, ^9^ Lee et al. found that in patients without diabetes, admission hyperglycemia was independently related to death and poor prognosis after ICH.^15^, ^18^, ^41^, ^42^, ^43^ However, Passero et al. found that DM is associated with a higher risk of death and poor prognosis.^42^, ^44^, ^45^ In most of the studies, hyperglycemia was usually measured as the admission blood glucose level without considering the pre‐onset blood glucose level or previous diabetes status. It must be noted that admission hyperglycemia could be caused by DM or SIH, which is a transient hyperglycemic condition caused by acute diseases, and some studies may not distinguish SIH from DM. In addition, patients with occult diabetes in some studies may be considered nondiabetic. All of the mentioned reasons may clutter the data and lead to misinterpretation of the results.

HbA1c is a reliable measure of the mean glucose concentration over the last 3‐ to 4‐months,^19^, ^20^ which could recognize occult diabetes. Chu et al analyzed HbA1c values and found that SIH was related to the risk of death and adverse prognosis after ICH.^13^, ^46^, ^47^ In this study, we combined the levels of HbA1c and admission glucose, along with DM history to distinguish between SIH and DM and classified patients into four groups: NDN, DN, DH, and SIH, as mentioned previously. We found that around 1/5 of patients (20.5%) had a history of DM, consistent with a previous report in which 15%–30% of patients with ICH had DM.^18^, ^48^, ^49^, ^50^, ^51^ Combined with the HbA1c results, 397 (28.9%) patients were d diagnosed with DM in this study, of whom 115 were diagnosed with occult DM (29.0%). Multivariate regression analysis revealed that SIH was associated with a higher risk of mortality at 30‐day and 90‐day after ICH, as well as pulmonary infection after ICH. However, DH did not increase the risk of poor outcomes or mortality. This finding indicates that SIH was more likely to be a risk factor for mortality and poor outcome of ICH than DM.

A previous study recruited 2039 patients with acute stroke, of which 533 (26.14%) were diagnosed with stroke‐associated pneumonia (SAP), and found that the stress hyperglycemia ratio (SHR) was significantly associated with the risk of SAP in patients without diabetes.^52^ Additionally, hyperglycemia reportedly leads to the excessive release of inflammatory factors, such as tumor necrosis factor‐α (TNF‐α), interleukin‐1 (IL‐1) and interleukin‐6 (IL‐6),^53^, ^54^ which were significant contributors to pulmonary infection.^55^, ^56^, ^57^, ^58^ Simultaneously, increased proinflammatory factors and immunosuppression caused by stroke promote and accelerate the occurrence of pulmonary infection.^59^, ^60^, ^61^ These findings shed light on the underlying mechanisms by which hyperglycemia increases post‐stroke pulmonary infections.

Evidence suggests that chronic hyperglycemia in patients with DM causes the body to form a self‐protection mechanism, preferentially down‐regulating glucose transporters (GLUT‐1 and GLUT‐3), allowing glucose to enter cells independently of insulin, thus reducing the acute fluctuation of glucose concentration and reducing endothelial cell apoptosis.^62^ This phenomenon may be a potential reason for the better outcome of hyperglycemia in patients with DM than in patients without diabetes after ICH.

This study had several limitations. Although the sample size was large, there are few patients with DH and SIH, and only Chinese patients were included. Additionally, due to medical insurance policies and costs, the proportion of patients for whom HbA1c was measured was low, which was the most important reason restricting the inclusion of patients. Furthermore, some other variables known to be associated with poor outcomes of ICH, such as hematoma expansion, were not analyzed in this study due to unavailability of data, which should be further studied in the future.

In conclusion, admission hyperglycemia is common in ICH patients and is associated with poor outcomes, of which SIH may be prioritized over DH to predict the risk of pulmonary infection and 30‐ and 90‐day death due to ICH.

## AUTHOR CONTRIBUTIONS

Shaoli Chen, Yan Wan, and Hongxiu Guo conducted data analysis and wrote the manuscript. Gang Li, Quanwei He, and Bo Hu designed the study and wrote the manuscript. All authors helped with the data collection and literature searches. All the authors have approved this version of the manuscript for publication.

## CONFLICT OF INTEREST

Dr. Bo Hu is an editorial board member of CNS Neuroscience and Therapeutics and is a co‐author of this article. To minimize bias, they were excluded from all editorial decision‐making related to the acceptance of this article for publication.

## Data Availability

Data are available on request from the authors.
